# A *dPIP5K* Dependent Pool of Phosphatidylinositol 4,5 Bisphosphate (PIP_2_) Is Required for G-Protein Coupled Signal Transduction in *Drosophila* Photoreceptors

**DOI:** 10.1371/journal.pgen.1004948

**Published:** 2015-01-29

**Authors:** Purbani Chakrabarti, Sourav Kolay, Shweta Yadav, Kamalesh Kumari, Amit Nair, Deepti Trivedi, Padinjat Raghu

**Affiliations:** 1 Inositide Laboratory, Babraham Institute, Cambridge, United Kingdom; 2 National Centre for Biological Sciences, TIFR-GKVK Campus, Bangalore, India; 3 Manipal University, Madhav Nagar, Manipal, Karnataka, India; 4 Department of Biological Sciences, Tata Institute of Fundamental Research, Colaba, Mumbai, India; New York University, United States of America

## Abstract

Multiple PIP_2_ dependent molecular processes including receptor activated phospholipase C activity occur at the neuronal plasma membranes, yet levels of this lipid at the plasma membrane are remarkably stable. Although the existence of unique pools of PIP_2_ supporting these events has been proposed, the mechanism by which they are generated is unclear. In *Drosophila* photoreceptors, the hydrolysis of PIP_2_ by G-protein coupled phospholipase C activity is essential for sensory transduction of photons. We identify dPIP5K as an enzyme essential for PIP_2_ re-synthesis in photoreceptors. Loss of *dPIP5K* causes profound defects in the electrical response to light and light-induced PIP_2_ dynamics at the photoreceptor membrane. Overexpression of dPIP5K was able to accelerate the rate of PIP_2_ synthesis following light induced PIP_2_ depletion. Other PIP_2_ dependent processes such as endocytosis and cytoskeletal function were unaffected in photoreceptors lacking *dPIP5K* function. These results provide evidence for the existence of a unique dPIP5K dependent pool of PIP_2_ required for normal *Drosophila* phototransduction. Our results define the existence of multiple pools of PIP_2_ in photoreceptors generated by distinct lipid kinases and supporting specific molecular processes at neuronal membranes.

## Author Summary

PIP_2_ has been implicated in multiple functions at the plasma membrane. Some of these require its hydrolysis by receptor-activated phospholipase C, whereas others, such as membrane transport and cytoskeletal function, involve the interaction of the intact lipid with cellular proteins. The mechanistic basis underlying the segregation of these two classes of PIP_2_ dependent functions is unknown; it has been postulated that this might involve unique pools of PIP_2_ generated by distinct phosphoinsoitide kinases. We have studied this question in *Drosophila* photoreceptors, a model system where sensory transduction requires robust phospholipase C mediated PIP_2_ hydrolysis. We find that the activity of phosphatidylinositol-4-phosphate 5 kinase encoded by *dPIP5K* is required to support normal sensory transduction and PIP_2_ dynamics in photoreceptors. Remarkably, non-PLC dependent functions of PIP_2_, such as vesicular transport and the actin cytoskeleton, were unaffected in dPIP5K mutants. Thus, dPIP5K supports a pool of PIP_2_ that is readily available to PLC, but has no role in sustaining other non-PLC mediated PIP_2_ dependent processes. These findings support the existence of at least two non-overlapping pools of PIP_2_ at the plasma membrane, and provide a platform for future studies of PIP_2_ regulation at the plasma membrane.

## Introduction

The detection and conversion of external stimuli into physiological outputs is a fundamental property of neurons and depends on intracellular signal transduction pathways. Phosphoinositides, the seven phosphorylated derivatives of phosphatidylinositol are key signalling molecules and of these the most abundant PIP_2_ has multiple roles in neurons. Several neuronal receptors (such as the metabotropic glutamate, growth factor and sensory receptors) transduce stimuli into cellular information using the hydrolysis of PIP_2_ by phospholipase C enzymes. Additionally, within the context of neuronal cell biology PIP_2_ has several roles including cytoskeletal function [1] [2] and several ion channels and transporters (eg: Kir, TRP and Na^+^/Ca^2+^ exchanger ) require PIP_2_ for their activity [3]. At the pre-synaptic terminal, a regulated cycle of PIP_2_ turnover is essential to regulate synaptic vesicle cycling. Thus PIP_2_ plays multiple roles at the plasma membrane of neurons; hence not surprisingly, changes in phosphoinositide metabolism have been linked to several inherited diseases of the human nervous system [reviewed in [4]]. Finally, one of the molecular targets of lithium, used in the treatment of bipolar disorders, is inositol monophosphatase a key regulator of PIP_2_ turnover in neurons [5].

Given the multiple functions of PIP_2_ at the plasma membrane, it is unclear if a common pool of PIP_2_ supports all these functions. Alternatively, if there are distinct pools, it is unclear how these are generated and sequestered on the nanoscale structure of the membrane. In principle, PIP_2_ can be generated by the activity of two classes of phosphatidylinositol phosphate kinase (PIPK) enzymes, designated PIP5K and PIP4K; PIP5K phosphorylates PI4-P at position 5 of the inositol ring, whereas PIP4K phosphorylates PI5-P at position 4 [[6]]. Although PIP4K and PIP5K synthesize the same end product, they are not functionally redundant [7] and studies of the mammalian enzymes has defined the molecular basis of substrate specificity [8]. Genes encoding PIP5K are present in all sequenced eukaryotes; however PIP4K appears to be a feature of metazoans; mammalian genomes contain three distinct genes for each of these two activities. However, the functional importance of these two classes of enzymes in generating plasma membrane PIP_2_ has remained unclear.

*Drosophila* photoreceptors are a well-established model for analyzing phosophoinositide signaling *in-vivo* [9]. In these cells, the absorption of photons is transduced into neuronal activity by G-protein coupled, phospholipase Cβ (PLCβ) mediated PIP_2_ hydrolysis [10]. Thus, during phototransduction, PIP_2_ needs to be resynthesized to match consumption by ongoing PLCβ activity. PIP_2_ turnover is tightly regulated in photoreceptors; mutants in molecules that regulate PIP_2_ turnover show defects in phototransduction [11]. However the role of PIPK enzymes in regulating PIP_2_ synthesis during phototransduction is unknown. In this study we have analyzed each of the three PIPK encoded in the *Drosophila* genome that could generate PIP_2_ in the context of phototransduction. Our analysis defines three pools of PIP_2_ supporting distinct molecular processes in photoreceptors.

## Results

### Multiple PIP kinases are expressed in the *Drosophila* eye

**Figure 1:**
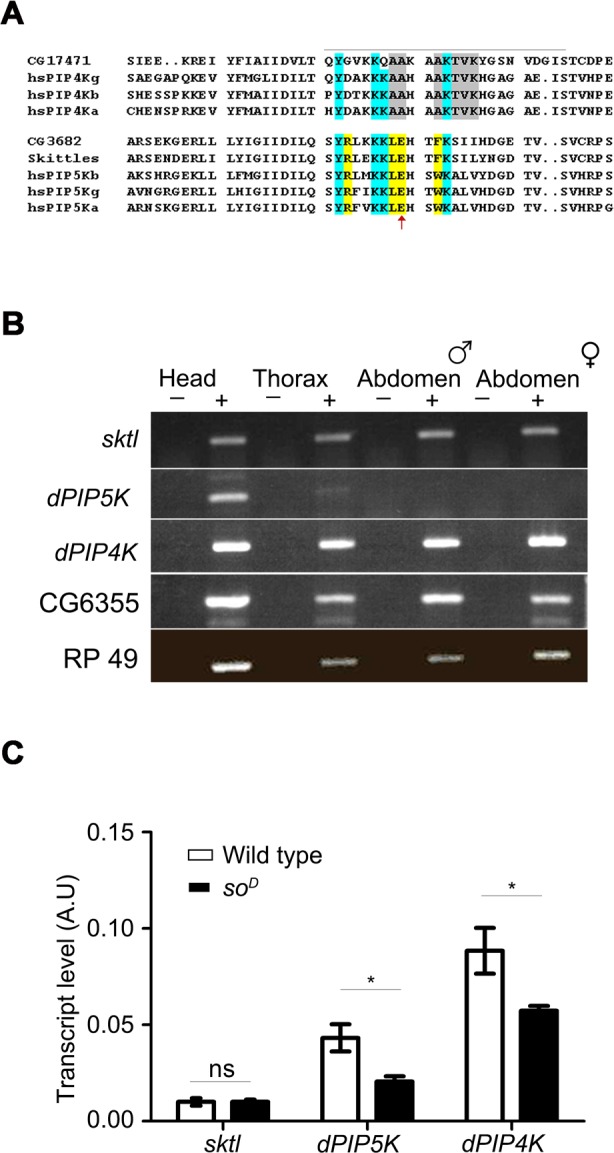
PIP kinase genes in *Drosophila* genome. A) Multiple alignment of the protein sequences of PIP4K and PIP5K genes. The sequence around the activation loop is presented with the region indicated by a grey line. Amino acid residues common between PIP5K and PIP4K proteins are marked in blue; residues unique to PIP5K are marked in yellow and unique residues for PIP4K in grey. The red arrow indicates the single residue described as responsible for the unique substrate specificity of PIP4K and PIP5K. Notations used for gene names are; hs-*Homo sapiens*; a-alpha; b-beta; g-gamma; CG17471-*Drosophila* PIP4K. B) RNA expression pattern of PIP kinases in various fly tissues: Qualitative RT (reverse transcription) PCR analysis with RNA extracted from various fly tissues. The tissue sources are labeled above the lanes. ‘+’ denotes +RT and ‘−’ denotes −RT. The corresponding gene names are indicated on the left side of the agarose gel picture. C) Comparative real time PCR analysis showing eye enrichment of dPIP5K and dPIP4K; the X-axis indicates gene names and the Y-axis represents transcript level expression in arbitrary units (A.U). White bars represent expression levels from cDNA samples of wild type fly heads and black bars represent samples from heads of *so^D^* (mutants that lack eyes). Values shown are the means ± S.D of three independent samples. p values between wild type and *so^D^* samples were determined using an unpaired *t*-test. The stars represent level of significance (***p< 0.001; **p< 0.01; *p< 0.05)

*In silico* analysis of the *Drosophila* genome sequence revealed that there are four distinct genes that encode open reading frames that include the Interpro domain IPR00002498 which is the “PIP kinase catalytic domain”. These include CG6355, CG3682, CG9985 and CG17471. Of these CG6355 encodes a FYVE domain containing protein that is the single ortholog of yeast *Fab1*, a protein with 1-phosphatidylinositol 3—phosphate 5 kinase activity [12][13]. CG17471 (*dPIP4K*) has recently been shown to encode a PIP4K activity that can generate PIP_2_ by 4 kinase activity using PI5P as a substrate [14]. The remaining two genes namely CG9985 (*sktl*) and CG3682 could encode putative PIP5K activity. *sktl* has been proposed to encode a *Drosophila* PIP5K [15][16]. CG3682 is an independent gene that also encodes a putative PIP5K activity. Previous studies have shown that the activation loop region of PIPKs contains specific residues that are conserved among PIP5K and are distinct from PIP4K enzymes [8]. A multiple alignment of PIP5K and PIP4K proteins from mammals and *Drosophila* reveals that *sktl* and CG3682 have activation loop residues that are highly diagnostic of those seen in mammalian PIP5K enzymes (Fig. 1A). Both SKTL and dPIP5K show high level of sequence similarity with all the isoforms of mammalian PIP5K. In catalytic domain the identity is more than 80%, whereas the overall sequence homology is from 55–65% with different mammalian isoforms. SKTL is ubiquitously expressed in all organs (Fig. 1B) suggesting its function in many/all cell types. In mammals this kind of expression pattern is evident for α and β isoforms of the PIP5K [17]. By contrast, the γ isoform of PIP5K is mostly expressed in neuronal tissues [18,19] an expression pattern recapitulated by dPIP5K. In addition *dPIP5K* has multiple splice variants with a conserved catalytic domain and variable C-terminal extensions [19]. The splicing pattern and protein isoforms of *dPIP5K* so generated as well as its expression pattern (enriched in the adult head Fig. 1B) recapitulates that seen for mammalian PIP5Kγ. The functional significance of the splice variants of *dPIP5K* remains to be established. In summary it is very likely that the *Drosophila* genome contains two genes that encode PIP5K activity namely *sktl* and CG3682. We have named CG3682 as *dPIP5K*. Thus, collectively there are three phosphoinositide kinases (PIPK), *sktl, dPIP5K* and *dPIP4K* all of which could generate PIP_2_.

In order to identify the PIPK that would generate PIP_2_ in adult *Drosophila* photoreceptors, we studied the expression of all three genes. We found that while *sktl* and *dPIP4K* were ubiquitously expressed in adult *Drosophila, dPIP5K* expression was mainly restricted to the head (Fig. 1B). All three genes were expressed in the *Drosophila* retina; while *sktl* RNA was present at very low levels and showed no eye-enrichment, *dPIP5K* and *dPIP4K*, showed some degree of enrichment in the eye (Fig. 1C).

### Loss-of-function mutants in *dPIP5K*

**Figure 2:**
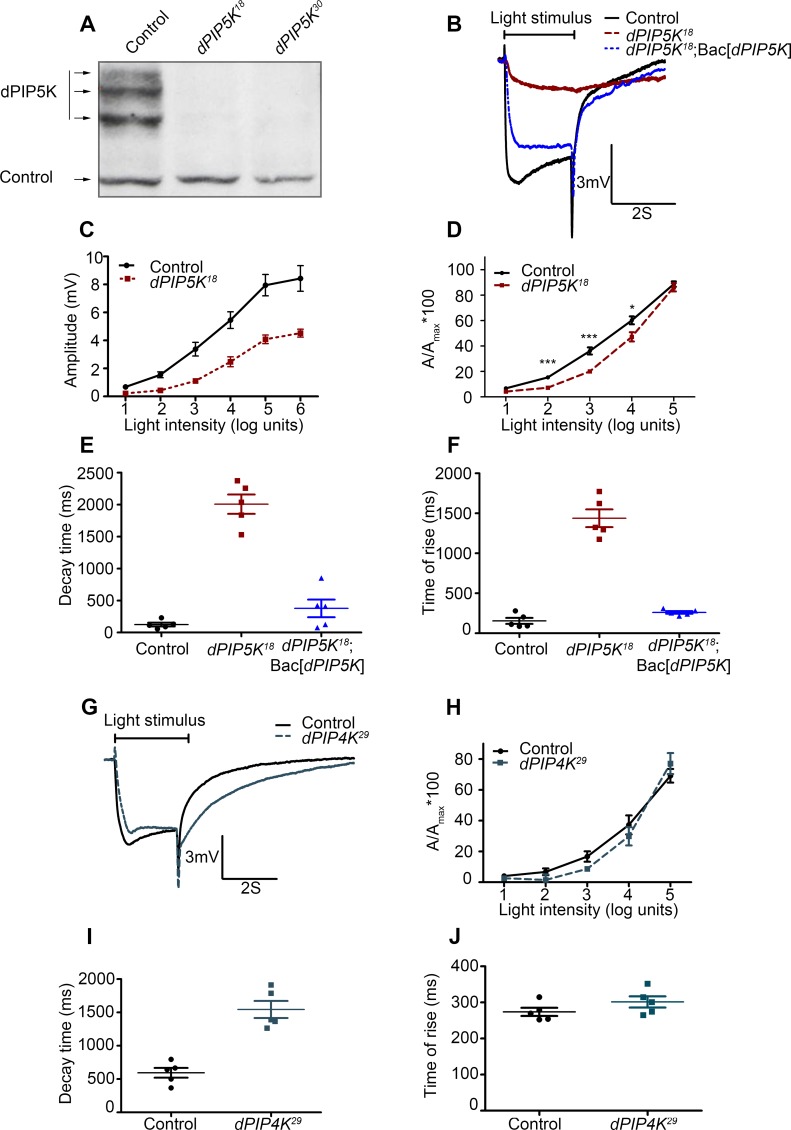
dPIP5K controls the light response in *Drosophila* photoreceptors. (A) Western blot of fly head extracts probed with dPIP5K specific antibody. The genotypes of the flies are labeled above each lane. The arrows indicate three bands corresponding to three different forms of dPIP5K detected with this antibody in wild type flies. All three bands are missing in both the knockout lines labeled as *dPIP5K^18^* and *dPIP5K^30^*. A protein loading control is shown labeled with a black arrow. (B) Representative ERG traces depicting the response of control and *dPIP5K^18^* photoreceptors to single 2s flash of green light of intensity 3 (cf. X-axis of Fig. 2D). The genotypes corresponding to each trace are indicated on the right side. Scale bar at the bottom shows the axis; X-axis represents time in seconds and Y-axis represents amplitude of the response in mV. The duration of the light pulse is indicated. Genotypes: Control-wild type; Mutant- *dPIP5K^18^*; Bac[*dPIP5K*] represents BAC clone containing the *dPIP5K* gene. (C) Graphical representation comparing the light response between control and *dPIP5K^18^*. The X-axis represents increasing light intensity in log units. The Y-axis represents peak amplitude of each response in mV. Error bars: Mean +/− S.D. (D) Intensity response function of the light response in control and *dPIP5K^18^* flies. Wild type and *dPIP5K^18^* flies with matched eye color are shown. The X-axis represents increasing light intensity in log units and Y-axis the peak response amplitude at each intensity normalized to the response at the maximum intensity. p values were determined using an unpaired *t*-test. The stars represent level of significance (***p< 0.001; **p< 0.01; *p< 0.05). Quantification of the decay time (time taken for the amplitude of the ERG response to reach 50% of its peak amplitude) (E) and the rise time of the ERG response (F) in control, *dPIP5K^18^* and *dPIP5K^18^*; Bac[*dPIP5K*]. (G) Representative light responses from control and *dPIP4K^29^* flies to single 2s flashes of green light. Scale bar at the bottom shows the axes; X-axis represents time in seconds and Y-axis represents amplitude of the response in mV. The duration of the light pulse is indicated. (H) Quantification of the intensity- response to light function from control and *dPIP4K^29^* flies. The X-axis represents light intensity in log units and Y-axis represents the peak amplitude of the response at a given intensity normalized to the response at the maximum intensity. (I) Quantification of the decay time (time taken for the amplitude of the ERG response to reach 50% of its peak amplitude) (J) and the rise time of the ERG response in controls and *dPIP4K^29^*.

In order to reveal the function of *dPIP5K in vivo*, a loss-of-function mutant was generated using ends-out homologous recombination [20]. This results in the insertion of a dominant selection marker (Pw^+^) flanked by multiple stop codons within the gene such that the kinase domain of dPIP5K was disrupted and the mutant allele should produce no protein. A total of eight independent knock-out alleles were isolated by following phenotypic markers, genetic mapping and molecular screening. Two of these namely *dPIP5K^18^* and *dPIP5K^30^* were studied in detail and are described in this study. All eight alleles were semi-lethal; very few homozygous mutant flies emerged as viable adults. Using a polyclonal antibody generated against a relatively unique C-terminal region of dPIP5K, we found that *dPIP5K^18^* and *dPIP5K^30^* were protein null alleles of *dPIP5K* (Fig. 2A).

In this study we also used a protein null allele of *dPIP4K* (*dPIP4K^29^)* that has already been described [14]. Homozygous deletions in *sktl* (eg: *sktl^Δ20^*) are larval lethal [15] and analysis using mitotic clones revealed that loss of *sktl* is also cell lethal in the developing eye. Thus for the analysis of *sktl* we have used an allelic combination *sktl^Δ20^ / +* and over expression of a kinase dead version of SKTL. These are not protein null alleles but represent the most severe alleles of *sktl* that give viable eyes.

### Abnormal light response in *dPIP5K^18/30^* photoreceptors

Since phototransduction in *Drosophila* involves rapid G-protein coupled PIP_2_ hydrolysis, it might be predicted that loss-of-function mutants in a PIPK that generates the PIP_2_ (which is the substrate for photoreceptor PLCβ) might also show a defective electrical response to light. We studied the response to light of mutants in all three genes encoding PIP kinases that can in principle generate PIP_2_, namely *dPIP5K, sktl* and *dPIP4K*. A widely accepted way to study the electrical response to light is the electroretinogram (ERG), an extracellular recording of light-induced electrical changes in the eye. Using ERG, we examined the electrophysiological responses of *dPIP5K^18^* photoreceptors to light. Since very few homozygous mutant adults were obtained, FLP/FRT mediated mitotic recombination was used to obtain mosaic animals in whom the whole eye was homozygous mutant for *dPIP5K^18^*[21,22]. ERGs were performed on day 0 (< 24hrs post-eclosion) flies. Characteristically, wild type photoreceptors respond with a large depolarization associated with on and off-transients. By contrast, photoreceptors from *dPIP5K^18^* produced a much smaller receptor potential in response to a stimulus of equivalent intensity associated with a very slow activation kinetics and response termination. Sample traces of voltage changes in response to a 2s stimulus of green light from wild type and mutants are shown (Fig. 2B). This phenotype was seen in all eight knock-out alleles of *dPIP5K* that we isolated. Responses in *dPIP5K^18^* displayed abnormal kinetics: the time of rise to the peak of the response was substantially prolonged (Fig. 2F) and the time for decay back to baseline following the end of the light stimulus was also increased (Fig. 2E). *dPIP5K^18^* photoreceptors did not display any on or off transients that represent synaptic activity at the first synapse between photoreceptors and the brain. An intensity response function analysis using flies of matched eye colour showed that *dPIP5K^18^* photoreceptors have a reduction in response sensitivity when measured over several log units of light intensity (Fig. 2C,D). Introduction of a genomic rescue transgene in *dPIP5K^18^* flies was able to largely correct the peak amplitude, response termination as well as restore both “on” and “off” transients completely (Fig. 2B, E, F). These results demonstrate that *dPIP5K* is required to support a normal electrical response to light in *Drosophila* photoreceptors.

By contrast light responses were unaffected in *dPIP4K^29^* [protein null mutant of *dPIP4K* [14]] photoreceptors (Fig. 2G,H). Although the amplitude of ERG responses from *dPIP4K^29^* look smaller than controls, this is because these flies are smaller than controls due to a growth defect during larval development [14]. However the kinetics of the light response were only marginally different in *dPIP4K^29^*. Finally we studied the most severe allele of *sktl* that gives rise to adult photoreceptors, namely *sktl^Δ20^/+* (S1A Fig.); these flies gave normal response to light. Further overexpression of a kinase dead version of *sktl* had no effect on the electrical response to light as measured by ERGs (S1B Fig.). Together these results suggest that *dPIP4K* and *sktl* are most likely dispensable for a normal electrical response to light in *Drosophila* photoreceptors.

### *dPIP5K^18^* photoreceptors show no major defects in ultrastructure or levels of transduction proteins

A number of mechanisms could account for the abnormal light response in *dPIP5K^18^* photoreceptors. PIP_2_ is known to be an allosteric regulator of a number of proteins involved in both vesicular transport as well as the cytoskeleton. Thus, loss of *dPIP5K* function might impact photoreceptor structure through defects in these processes and the abnormal light response may be a consequence of abnormal ultrastructure as seen in the case of mutants such as *rdgA* and *rdgB* [23].

**Figure 3:**
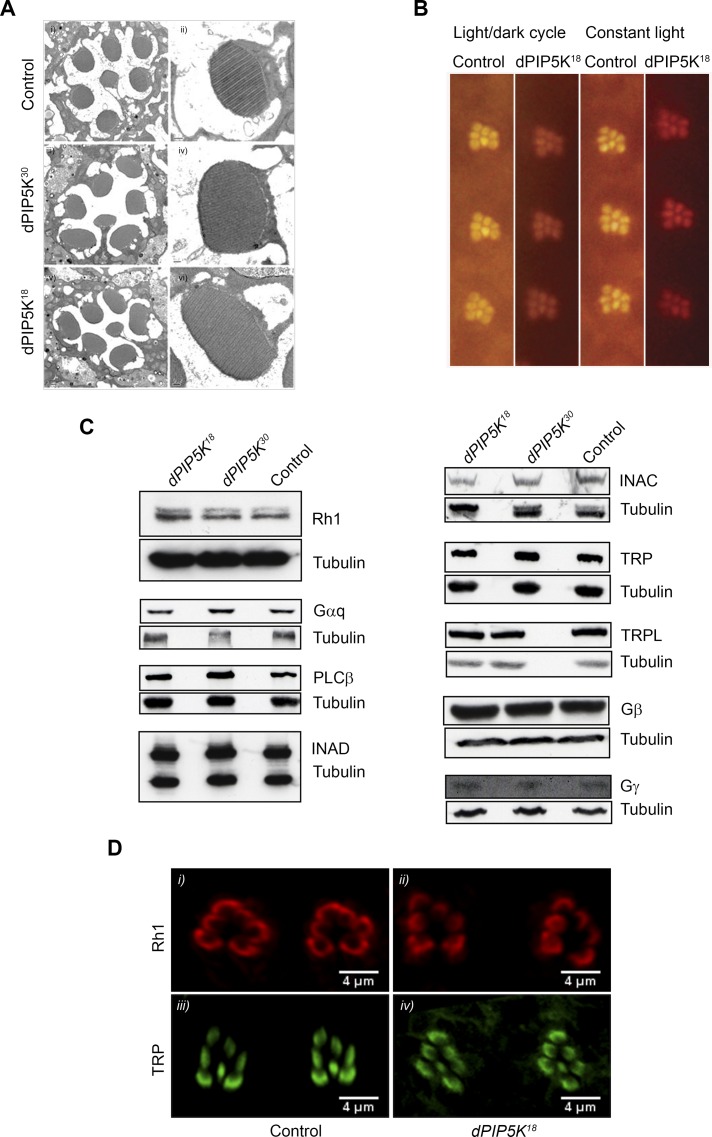
*dPIP5K^18^* photoreceptors have normal ultrastructure and unaltered levels of transduction proteins. (A) TEM images showing the ultrastructure of control (i-ii), *dPIP5K^30^* (iii-iv) and *dPIP5K^18^* (v-vi). The cross sectional view of a single ommatidium (i, iii, v) and a high magnification view of a single rhabdomere (ii, iv, vi) are shown for each genotype. (B) Optical neutralization images of *dPIP5K^18^* retinae showing normal rhabdomere ultrastructure in flies grown in a 12h L/D cycle as well as in 24 hrs constant light. Images shown are from flies aged nine days post-eclosion. (C) Western blot analysis of head extracts from wild type, *dPIP5K^18^*, *dPIP5K^30^* probed with antibodies to each of the major phototransduction proteins. The antibodies used are indicated at the right side of each panel. Tubulin is used as loading control for each set of blots. (D) Single optical transverse sections of a control and *dPIP5K^18^* retina probed with antibodies to Rhodopsin (Rh1) (i, ii) and TRP (iii, iv).

To test this hypothesis, we studied photoreceptor ultrastructure using transmission electron microscopy (TEM). This revealed that photoreceptors R1-R7 from 0 day old flies were normal in *dPIP5K^18^*(Fig. 3A). Microvilli were completely intact and showed no vesiculation or blebbing and only minimal defects were seen at the base of the microvilli; however these changes did not increase with age or illumination and rhabdomere structure remained completely intact (Fig. 3B).

A reduced light response could also result from reduction in the levels of key proteins required to support the phototransduction cascade. Thus the abnormal light response in *dPIP5K^18^* photoreceptors could be due to altered levels of any one of the key proteins required for phototransduction such as Gq and Rh1. Gαq^1^, a severe hypomorph of *dGq* expressing less than 1% of the wild type protein levels shows more than 1000-fold reduction in sensitivity to light [24]and *ninaE* mutants characterized by large decrease in the level of Rh1 also show reduced sensitivity to light. Alternatively, the abnormal ERG could also be the consequence of reductions in the level of proteins like NORPA, TRP, INAD which act downstream of photon absorption to generate a normal electrical response to light. This led us to check the protein level of all the key components of the phototransduction cascade in *dPIP5K^18^*. Western blot analysis (Fig. 3C) showed that neither the levels of Rh1, Gαq, Gβ and Gγ, nor the levels of NORPA, TRP, TRPL, INAD and INAC were appreciably reduced compared to controls. This result suggests that the abnormal light response observed in *dPIP5K^18^* is not due to reduced levels of any of the key protein required for phototransduction. Further the subcellular localization of Rh1 and TRP were also studied and found to be not different between control and *dPIP5K^18^* (Fig. 3D). Together, these findings suggest that ultrastructural defects or changes in the levels and localization of the major transduction proteins cannot explain the abnormal electrical response in *dPIP5K^18^* photoreceptors.

### *dPIP5K* regulates light induced PIP_2_ dynamics in photoreceptors

**Figure 4:**
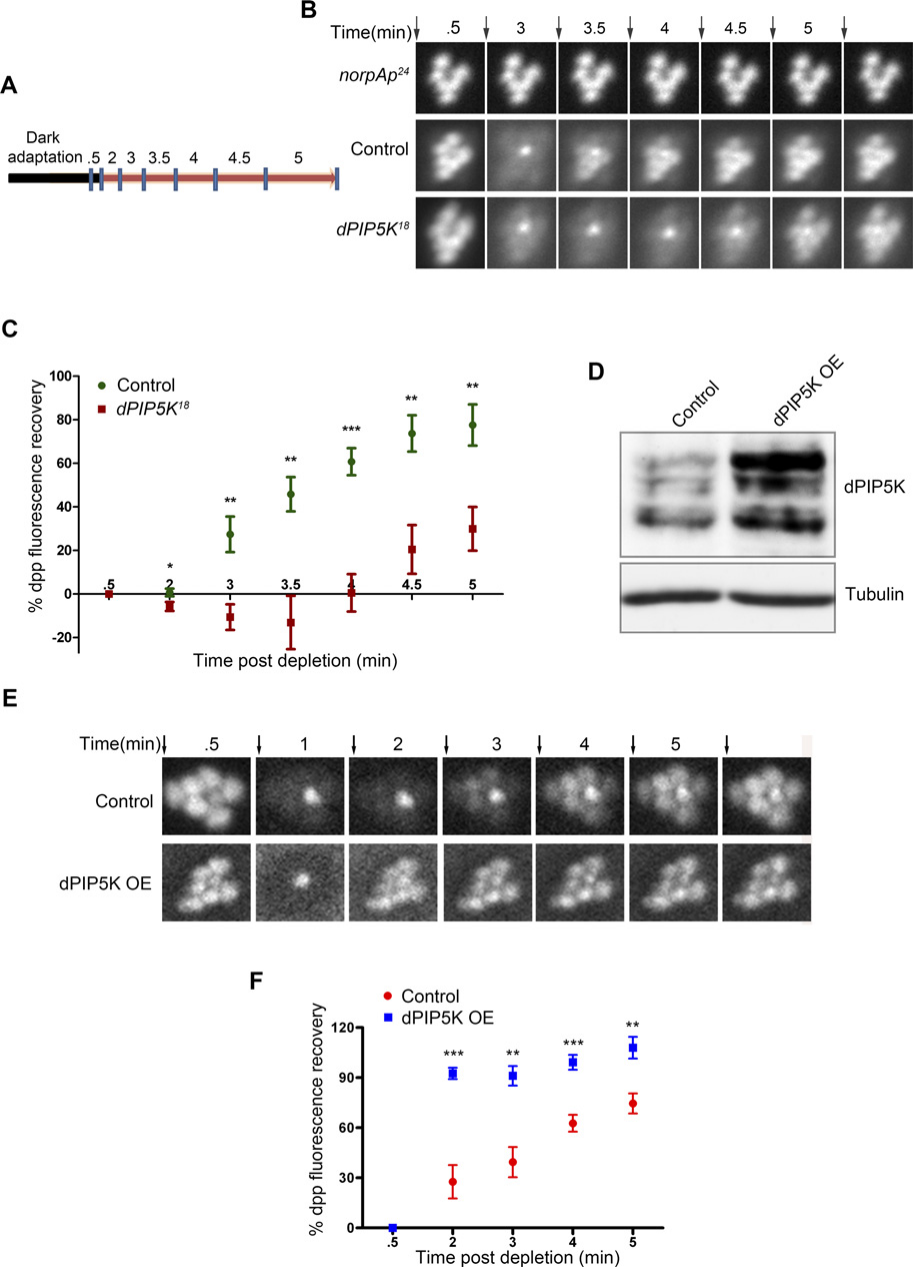
dPIP5K controls PIP_2_ dynamics in *Drosophila* photoreceptors. (A) Diagrammatic representation of the experimental protocol used to study PIP_2_ dynamics in the intact eye. The blue symbols indicate the time of image acquisition. The color of the bar indicates the light condition at which the fly was kept during the experiment. Black color indicates total dark and red indicates in red light illumination. The time points are labeled above the bar in minute. The detailed experimental procedure is discussed in material and methods section. (B) Fluorescent deep pseudopupil (dpp) imaging to study PIP_2_ dynamics using flies expressing the PIP_2_ biosensor (see main text for details). The time scale of the imaging is indicated on the top of each panel. Arrows indicate the timing of a 90 ms flash of blue light used for imaging the dpp. Images were acquired from control, *dPIP5K^18^* and *norpA^P24^*. The genotypes used for the image acquisition are labeled at the left of the image panel. *norpA^P24,^* which is a protein null mutant of PLCβ, is used to show the dependence of dpp dynamics on PLCβ activity. (C) Quantitative representation of PIP_2_ dynamics. X-axis represents time in minutes between the depleting flash of blue light and the next image acquired. During this period eyes were illuminated in red light. Y-axis represents the level of fluorescence represented as a % of the value in the initial image. Error bars represents mean +/− S.D from five flies. p values were calculated using an unpaired *t*-test. The stars represent level of significance (***p< 0.001; **p< 0.01; *p< 0.05) (D) Western blot from head extracts depicting the level of dPIP5K protein expression in wild type flies and those overexpressing dPIP5K. The blot was probed with antibody to dPIP5K. Tubulin was used as loading control. (E) Representative images of dpp imaging in control flies and those overexpressing dPIP5K. (F) Quantification of PIP_2_ dynamics in flies overexpressing *dPIP5K* compared to controls. X-axis represents time in minutes and Y-axis represents the level of fluorescence represented as a % of the value in the initial image. Error bars represents mean +/− S.D from five flies. p values were determined using an unpaired *t*-test. The stars represent level of significance (***p< 0.001; **p< 0.01; *p< 0.05).

dPIP5K is a PIP kinase that is predicted to convert PI4P into PIP_2_. To test its requirement in regulating the dynamics of light induced PIP_2_ turnover at the rhabdomeral membrane we used a live fly preparation in which a fluorescent biosensor consisting of the PH domain of PLCδ fused to GFP (hereafter called PIP_2_ biosensor) is expressed in photoreceptors. When eyes are illuminated with bright light (λ_max_ 488 nm) high rates of PLC activation result in the hydrolysis of PIP_2_ and as a consequence the fluorescent biosensor is detached from the membrane and diffuses out of the microvillar cytoplasm resulting in the loss of the fluorescent pseudopupil signal. Under red light illumination that converts metarhodopsin to rhodopsin thus terminating PLCβ activity, PIP_2_ levels recover as a consequence of ongoing PIP_2_ resynthesis and the PIP_2_ biosensor signal in the pseudopupil recovers (Fig. 4A,B). The kinetics of the fluorescent pseudopupil are blocked in *norpA^P24^* which lacks appreciable PLCβ activity (Fig. 4B). Using this approach we studied the kinetics of light induced PIP_2_ turnover in *dPIP5K^18^* and compared it to wild type controls. This analysis revealed a clear delay in the kinetics of PIP_2_ resynthesis in *dPIP5K^18^* compared to wild type controls and suggests that dPIP5K activity is required to support normal PIP_2_ resynthesis following phototransduction (Fig. 4B).

We also tested the effects of overexpressing dPIP5K in adult photoreceptors; the level of protein overexpression was established by Western blots of retinal extracts using an antibody to dPIP5K (Fig. 4D). Overexpression of dPIP5K in photoreceptors resulted in a marked acceleration in the recovery of the PIP_2_ biosensor following stimulation with light (Fig. 4E, F). This result implies that dPIP5K is able to regulate the rate of PIP_2_ resynthesis following illumination in photoreceptors.

### *dPIP5K* does not support cytoskeletal function at the microvillar membrane

**Figure 5:**
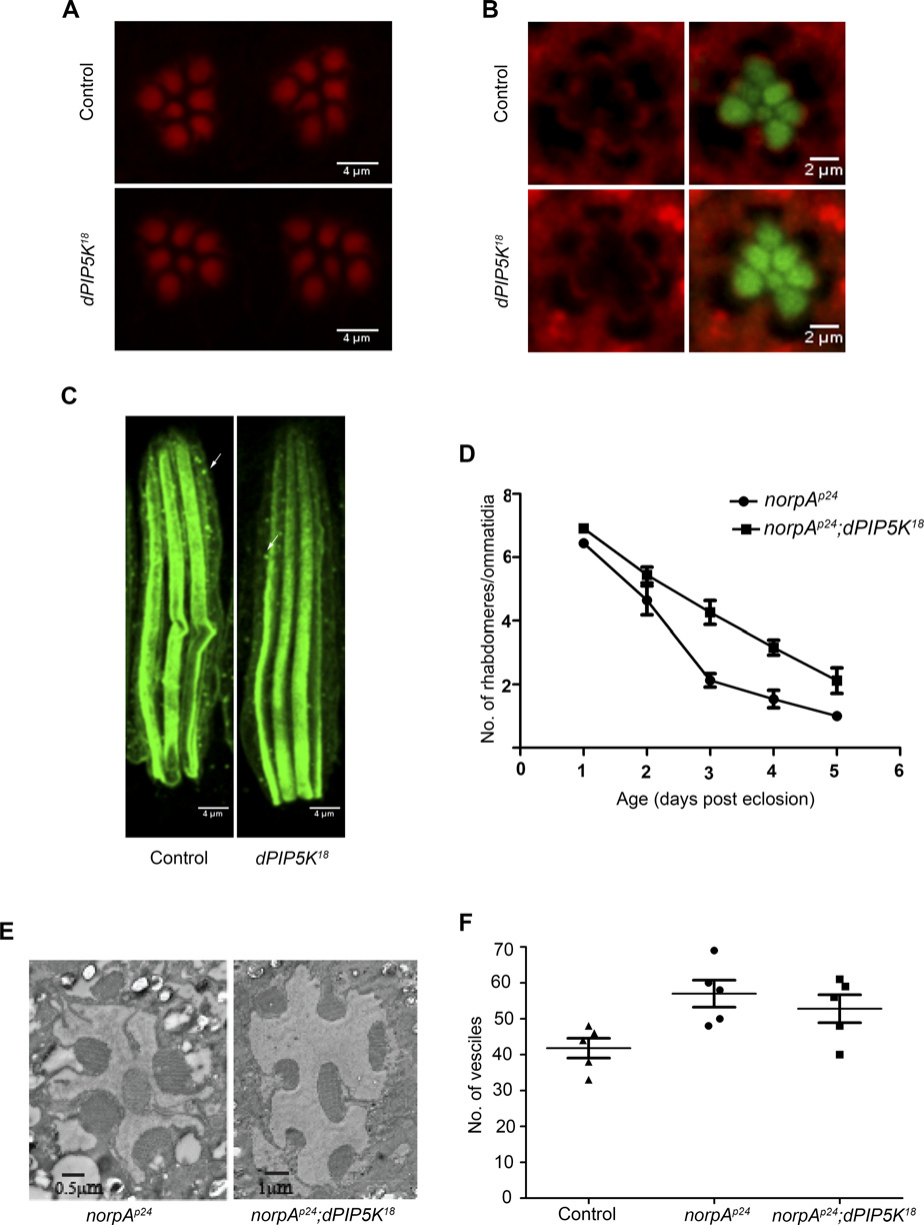
dPIP5K is not required to support cytoskeleton function and dynamin mediated endocytosis in adult photoreceptors. (A) Confocal images of phalloidin stained retinae from control and *dPIP5K^18^* photoreceptors showing normal staining of the rhabdomeres. (B) Confocal images of retinae stained with a p-moesin specific antibody from control and *dPIP5K^18^*. Red pseudo color represents p-moesin staining and green marks the rhabdomeric region stained with phalloidin. (C) Confocal images showing longitudinal sections from retinae stained with an antibody to Rh1. The arrows indicated Rhodopsin Loaded Vesicle (RLV) involved in Rh1 endocytosis and recycling. (D) Rate of photoreceptor degeneration in *norpA^P24^* and *norpA^P24^; dPIP5K^18^* monitored using optical neutralization. The flies were reared in continuous light at 2300 lux. The X-axis represents age of the flies and the Y-axis represents number of rhabdomere visualized in each ommatidium. Error bars represent mean +/− S.D from 50 ommatidia taken from at least five flies. (E) Representative transmission electron micrographs of retinae from *norpA^P24^* and *norpA^P24^; dPIP5K^18^* flies showing the degree of preservation of ultrastructure. Images shown are from flies that are three days old gown under the same illumination conditions as for panel D. (F) Quantitative representation of Rhodopsin Loaded Vesicles (RLVs) in control, *norpA^p24^* and *norpA^p24^;dPIP5K^18^*. The genotype of the fly is shown in the X-axis and Y-axis represents the count of RLVs.

PIP5K has been shown to have a role in actin remodeling in both yeast and mammalian systems [25]. In *dPIP5K^18^*, photoreceptor ultrastructure was largely normal by TEM analysis and growing flies under conditions of bright light illumination did not result in disruption of microvillar ultrastructure. This suggests that the actin cytoskeleton is unaffected by the absence of dPIP5K activity. Further, phalloidin staining suggested that the actin cytoskeleton was largely unaffected in *dPIP5K^18^* (Fig. 5A). The **E**zrin/**R**adixin/**M**oesin (ERM) family of proteins are regulated by PIP_2_ and act as cross-linkers between cortical actin and plasma membrane thus playing a key role in maintaining membrane projections, such as microvilli and filopodia [26][27]. ERM proteins are regulated by PIP_2_ and in the presence of PIP_2_ the active, phosphorylated form of the protein is attached to the membrane; on PIP_2_ hydrolysis the proteins are dephosphorylated and the inactive form is released to cytosol [28]. *Drosophila* has only a single ERM protein, dMoesin, which is required for morphogenesis and maintenance of microvillar structure in photoreceptors. Moesin localizes to the base of the rhabdomere in wild-type flies in the dark [29], but is dephosphorylated and translocates to the cytosol under bright illumination. Using immunolabelling we studied the distribution of p-Moesin in photoreceptors and found this to be no different between controls and *dPIP5K^18^*(Fig. 5B). Collectively these findings suggest that dPIP5K activity is not required to support cytoskeletal function in adult *Drosophila* photoreceptors.

### *dPIP5K* is not required to support rhodopsin turnover at the microvillar plasma membrane

In *Drosophila* photoreceptors during illumination rhodopsin, the G-protein coupled receptor for light is endocytosed into a vesicular compartment called rhodopsin loaded vesicles (RLV) [30], a key compartment in the turnover of rhodopsin. PIP_2_ is an important regulator of multiple steps in the endocytic cycle [31]. To test if *dPIP5K* supports the synthesis of the PIP_2_ pool regulating endocytosis, we observed the number of RLV in photoreceptors from *dPIP5K^18^* photoreceptors; these were very similar to those in wild type (Fig. 5C). We also tested the effect of *dPIP5K^18^* on the retinal degeneration phenotype of *norpA* this has previously been shown to depend on endocytosis. *norpA* mutants undergo light dependent retinal degeneration due to the accumulation of excessive amounts of metarhodopsin-arrestin2 (Arr2-Rh) complexes stabilised at the microvillar membrane [32,33]. This process has been shown to depend on clathrin-mediated endocytosis, a process requiring a number of PIP_2_ dependent steps. To test if this was affected in *dPIP5K^18^*, we generated double mutants of *norpA^P24^;dPIP5K^18^* and studied the time-course of light dependent retinal degeneration in comparison with *norpA^P24^* alone. If the PIP_2_ pool produced by *dPIP5K* was required to mediate endocytosis then one might expect reduced endocytosis of Arr2-Rh complexes in *dPIP5K^18^* photoreceptors thus suppressing the retinal degeneration phenotype of *norpA^P24^*. Such an effect is seen when dynamin function is removed using the *shibire* mutant that results in suppression of degeneration in *norpA* [32]. However we found that light dependent retinal degeneration was not suppressed in *norpA^P24^; dPIP5K^18^*, although the time course of degeneration was marginally slower (Fig. 5D, E). Additionally the number of RLVs in *norpA^P24^* and *norpA^P24^; dPIP5K^18^* were not significantly different (Fig. 5F). These results suggest that dPIP5K function is not required to support the endocytic turnover of rhodopsin.

### Subcellular localization of dPIP5K

**Figure 6:**
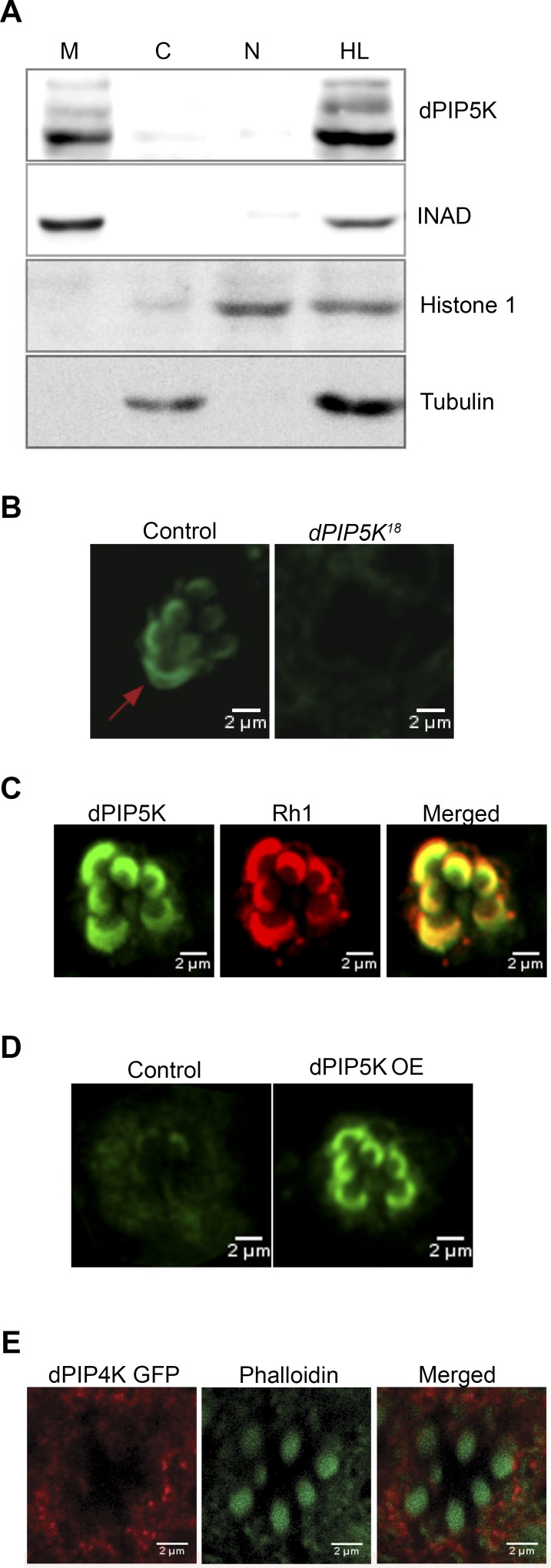
Subcellular localization of different PIPKs in adult photoreceptors. (A) Western blot showing localization of dPIP5K in different sub-cellular fractions prepared from adult Drosophila heads. Fractions shown are: HL-total head lysate; C-cytoplasm; N, nuclear; M-microsomal/membrane. An antibody to INAD is used as a membrane marker. Histone 1 has been used as a nuclear marker and Tubulin as a marker for cytosol. (B) Confocal image showing the distribution of endogenous dPIP5K as detected by a polyclonal antibody from wild type and *dPIP5K^18^* photoreceptors. The enrichment of dPIP5K staining at the rhabdomere membrane (arrow) in wild type is missing in *dPIP5K^18^*. (C) Double staining experiments on wild type retinae showing the co-localization of dPIP5K (green) with Rh1 (red). (D) Localization of dPIP5K overexpressed from its endogenous genomic locus. Confocal image from retinae of control flies and those overexpressing dPIP5K using Rh1-GAL4. The protein is shown localized to the rhabdomere membrane. (E) Confocal image showing localization of overexpressed dPIP4K in adult *Drosophila* photoreceptors. Phalloidin staining marking the rhabdomeres shown in green and dPIP4K localization detected by antibody labeling shown in red. Merged image shows that dPIP4K is excluded from the rhabdomeres.

Given our prediction that dPIP5K generates PIP_2_ that is used as a substrate for light induced PLCβ activity, it is likely that the enzyme is localized to the microvillar plasma membrane where PLCβ is localized. Initial fractionation experiments showed that almost all of the dPIP5K is localized to a membrane fraction (Fig. 6A) where it co-fractionates with key phototransduction proteins such as INAD. We attempted to establish the localization of dPIP5K expressed at endogenous levels using immunocytochemistry; under these conditions the dPIP5K antibody was able to detect the protein localized at the microvillar membrane (Fig. 6B); this was abolished in retinae from *dPIP5K^18^* photoreceptors that are protein null alleles for this gene. Double labeling experiments showed that dPIP5K co-localizes with Rh1 at the microvillar membrane (Fig. 6C). We exploited a genetic tool [34] that allowed us to elevate the expression level of untagged endogenous dPIP5K in photoreceptors, expressed from the endogenous gene locus. Under these conditions too, we found dPIP5K clearly localized to the microvillar membrane (Fig. 6D). By contrast a dPIP4K::GFP transgene was excluded from the microvillar plasma membrane (Fig. 6E).

### Interaction of dPIP5K with *rdgB*

**Figure 7:**
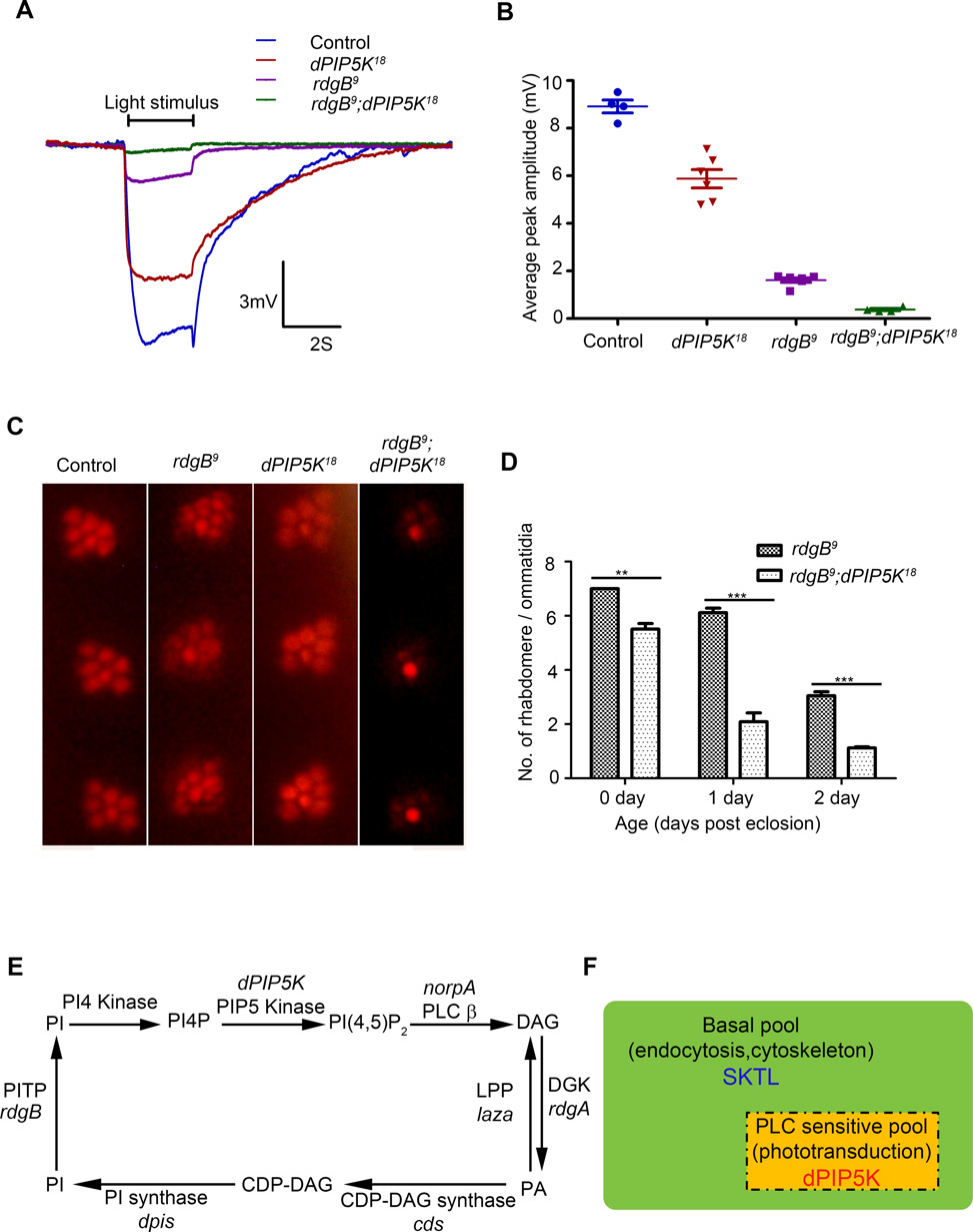
dPIP5K is required to support *rdgB* dependent function in photoreceptors. (A) Representative ERG traces depicting the response of control, *dPIP5K^18^*, *rdgB^9^* and *rdgB^9^; dPIP5K^18^*. Responses to single 2s flash of green light (intensity 5 cf. X-axis of Fig. 2D) are depicted. The genotypes corresponding to each trace are indicated on the top of the graph. Scale bar at the bottom shows the axis; X-axis represents time in seconds and Y-axis represents amplitude of the response in mV. The duration of the light pulse is indicated. (B) Comparison of maximum amplitude of the light response among control, *dPIP5K^18^, rdgB^9^* and *rdgB^9^; dPIP5K^18^*. Y-axis represents mean +/− S.D of the peak amplitude from five flies. (C) Representative optical neutralization images from control, *rdgB^9^*, *dPIP5K^18^* and *rdgB^9^; dPIP5K^18^* showing the exacerbation of degeneration in *rdgB^9^; dPIP5K^18^* compared to *rdgB^9^, dPIP5K^18^* alone does not show any degeneration. The representative images shown are collected from one-day-old flies maintained in L/D cycle (900 lux). (D) Quantification of the effect of *dPIP5K^18^* loss of function on photoreceptor structure in *rdgB^9^*. (E) Schematic diagram showing the light induced PIP_2_ cycle in *Drosophila* photoreceptors. Genes encoding a given enzyme activity where identified are marked in italics. Notations used: PI(4,5)P_2_-phosphatidylinositol 4,5 bisphosphate, *norpA*- no receptor potential A, PLCβ-phospholipase C beta, DAG- diacylglycerol, DGK- diacylglycerol kinase, *rdgA- r*etinal *d*egeneration *A*, *laza*-lipid phosphate phosphohydrolase (PA phosphatase), PA-phosphatidic acid, CDP-DAG- Cytidine diphosphate diacylglycerol; CDS CDP-DAG synthase, PI- phosphatidylinositol, *rdgB- r*etinal *d*egeneration *B*, PITP- phosphatidylinositol transfer protein, PI(4)P- phosphatidylinositol 4-phosphate (F) Schematic representation of the pools of PIP_2_ in *Drosophila* photoreceptor membranes. Representations are only semi-quantitative. The total pool of PIP_2_ in the plasma membrane is shown bounded by the solid black line. The PLC sensitive PIP_2_ pool sensitive to light induced PLC activity is shown in the rectangle bounded by the broken/dashed line indicating that PLC will likely also use a non-dPIP5K dependent pool. The basal PIP_2_ pool is indicated in green. The major function of each pool is indicated. Enzymes responsible for the synthesis of each pool are marked.

Photoreceptors of the *Drosophila rdgB* mutant show defects in the electrical response to light as well as light dependent degeneration. *rdgB* encodes a large multi domain protein including an N-terminal phosphatidylinositol transfer protein (PITP) domain [reviewed in [35]]. *In vitro* the PITP domain can bind and transfer phosphatidylinositol (PI) between two membrane bound compartments and it is presumed, though not demonstrated that the PI delivered to the acceptor compartment is the substrate for phosphorylation by PIPKs that generate phosphorylated versions of PI. In the case of PIP_2_ this would include the sequential phosphorylation of PI and PI4P by PI4K and PIP5K respectively. Although the precise molecular function of RDGB in photoreceptors in unknown, it has previously been shown that *rdgB* mutant photoreceptors have a defect in restoring the level of microvillar PIP_2_ following transduction triggered by a bright flash of light [36]. Thus *rdgB* mutants represent an opportunity to test the importance of a potential PIP5K that might generate microvillar PIP_2_ required for phototransduction. To test the relevance of dPIP5K in generating PIP_2_ required for G-protein coupled PLCβ activity, we generated photoreceptors that are double mutant *rdgB^9^; dPIP5K^18^*; importantly we used the *rdgB^9^* allele that is a strong hypomorph and expresses a small amount of this protein and therefore has a residual response to light. We compared the light response of *rdgB^9^* photoreceptors with those of *rdgB^9^; dPIP5K^18^* (Fig. 7A). Under similar conditions, while *rdgB^9^* photoreceptors have peak ERG amplitudes of ca. 1.5 mV (Fig. 7A), *rdgB^9^; dPIP5K^18^* photoreceptors respond with a amplitude of only 0.4 mV (Fig. 7B). This observation suggests that *dPIP5K* function is required to support the residual light response in *rdgB^9^* photoreceptors. We also studied a second phenotype of *rdgB^9^* namely light dependent retinal degeneration and found that, *rdgB^9^; dPIP5K^18^* photoreceptors degenerated faster than *rdgB^9^* alone (Fig. 7C,D). By contrast loss of *dPIP4K* or *sktl* did not exacerbate the electrical response to light or the retinal degeneration phenotype of *rdgB^9^*.

## Discussion

The hydrolysis of PIP_2_ by PLC in response to receptor activation is a widespread mechanism of signalling at the plasma membrane. In some cells such as neurons, activation of cell surface receptors by neurotransmitter ligands (e.g glutamate, Ach) or sensory stimuli triggers high rates of PLC activation and rapid consumption of PIP_2_. Under these conditions, it is essential that levels of PIP_2_, the substrate for PLC are maintained as failure to do so would likely result in desensitization. In mammalian cells, multiple classes of PIPK, the enzymes that resynthesize PIP_2_ have been described; yet the contribution of these enzymes to PIP_2_ resynthesis following PLC activation during cell signalling *in vivo* remains unclear. Broadly two classes of PIPK can synthesize PIP_2_ have been described; PIP5K that phosphorylates PI4P at position 5 [37] or PIP4K that can phosphorylate PI5P at position 4 [38]. In this study we have analyzed the consequence of loss of each of these two classes of PIPK to resynthesis following PLC mediated PIP_2_ depletion during *Drosophila* phototransduction. Loss of *dPIP5K* function results in profound defects in the light activated electrical response as well as slower recovery of plasma membrane PIP_2_ levels. Conversely overexpression of dPIP5K was able to substantially accelerate the recovery of PIP_2_ levels following stimulation with a bright flash of light. We found that dPIP5K is localized to the microvillar plasma membrane, the site at which PIP_2_ needs to be produced to support ongoing light induced PLC activity. Finally, we found that loss of dPIP5K enhances the ERG defect in a hypomorphic allele of *rdgB*, a gene with a well-established defect in the response to light. Collectively these observations strongly suggest that dPIP5K activity underlies the conversion of PI4P to PIP_2_ at the microvillar membrane where it is then available as a substrate for light induced PLCβ activity (Fig. 7E). By contrast loss of the only PIP4K enzyme in the *Drosophila* genome has minimal effects on phototransduction and this enzyme is not targeted to the microvillar plasma membrane. Our findings also imply that dPIP4K activity (and hence the conversion of PI5P into PIP_2_) is dispensable for maintaining PIP_2_ levels during *Drosophila* phototransduction. This is consistent with a previous study which found no reduction in the levels of PIP_2_ in flies lacking dPIP4K function [14]. Our observations validate the conclusion from biochemical studies in mammalian cells that the levels of PI5P are substantially lower than those of PIP_2_ and hence it is unlikely to be the source of the majority of PIP_2_ in cells [38]. The identity of the PI4K isoform that generates the substrate, PI4P used by dPIP5K remains unknown although a recent study in mammalian systems suggests that PI4KIIIα is likely to be the relevant isoform [39].

Although the ERG response is severely affected in *dPIP5K^18^*, it is not abolished as seen in null mutants of PLCβ (*norpA)* that are not able to hydrolyse PIP_2_. Additionally, the resting levels of PIP_2_ as detected by the PIP_2_ biosensor are comparable to wild type and following a bright flash of light that depletes PIP_2_, its levels do recover albeit at a slower rate than in wild type photoreceptors. Given that *dPIP5K^18^* is a protein null allele, these observations imply that there must be a second pool of PIP_2_ in *dPIP5K^18^* cells that is able to support phototransduction and microvillar PIP_2_ re-synthesis albeit with lower efficiency (Fig. 7F). This second pool of PIP_2_ is likely available with low efficiency for PLC activity in the absence of the dPIP5K dependent pool thus accounting for the residual light response and observed PIP_2_ dynamics in *dPIP5K^18^* photoreceptors.

We found that the ultrastructure of *dPIP5K^18^* photoreceptors was essentially normal. This was particularly surprising given that in addition to phototransduction, PIP_2_ at the microvillar membrane is also expected to regulate multiple processes required to maintain normal microvillar structure including dynamin dependent endocytosis [40][32] as well as cytoskeletal function [41]. However, using multiple readouts we found that molecular readouts of endocytosis and cytoskeletal function were unaffected in *dPIP5K^18^* photoreceptors (Fig. 5). These observations imply that the PIP_2_ required for these processes is not dependent on dPIP5K activity; rather PIP_2_ generated by a separate PIPK supports these processes. Thus far, dPIP4K has not been detected on the microvillar plasma membrane, *dPIP4K^29^* photoreceptors show normal ultrastructure on eclosion and do not undergo light dependent microvillar degeneration; thus dPIP4K is unlikely to be the critical enzyme that generates the PIP_2_ required to support dynamin dependent endocytosis, p-Moesin localization or phototransduction. The *Drosophila* genome encodes an additional PIP5K activity, *sktl* that is expressed at low levels in the adult retina but is localized to both the microvillar and basolateral membrane and hence could synthesize PIP_2_ at both these locations. Complete loss of *sktl* function is cell lethal and overexpression of *sktl* in developing photoreceptors results in a severe block in rhabdomere biogenesis [42] whereas overexpression of *sktl* results in light dependent retinal degeneration in post-development photoreceptors. These findings presumably reflect an essential and non-redundant role for SKTL in supporting fundamental PIP_2_ dependent cellular processes such as endocytosis and cytoskeletal function that are not dependent on PIP_2_ hydrolysis by PLC. This model is consistent with the cell-lethal phenotype of photoreceptors that are null for *sktl* and previous studies showing a role for *sktl* in supporting cytoskeletal function and endocytosis in other *Drosophila* tissues and processes such as spermiogenesis [43] and oogenesis [44].

Collectively, our observations imply that there are at least two pools of PIP_2_ in photoreceptors; one generated by dPIP5K that is required to support a normal electrical response to light but is dispensable for non-PLC dependent functions of PIP_2_ in photoreceptors and another that is generated by enzymes other than dPIP5K (most likely SKTL) that is also capable of supporting PIP_2_ synthesis during the light response albeit with reduced efficiency. In summary the PIP_2_ pool synthesized by dPIP5K is unique in that it is required for a normal light response and apparently dispensable for other PIP_2_ dependent functions/processes. It also reflects the existence of distinct/segregated pools of PIP_2_ on the same microvillar plasma membrane that are maintained by distinct kinases.

A number of previous studies have shown that in multiple eukaryotic cell types, plasma membrane PIP_2_ levels are remarkably stable, undergoing transient fluctuations despite ongoing PLC mediated PIP_2_ hydrolysis [36,45–47]. However the reasons for this remarkable finding have remained unclear although pharmacological studies have suggested the importance of PIP_2_ resynthesis in this process [47,48]. One potential explanation for this idea is the existence at the plasma membrane of two pools of PIP_2_, a larger but less dynamic pool of that is not normally accessed by PLC and supporting non-PLC dependent functions of this lipid and a second, quantitatively smaller but more dynamic pool that is the substrate for PLC activity. What underpins such pools of PIP_2_? The existence of separate enzymes that generate unique pools of PIP_2_ has been previously suggested but there have been limited experimental studies to support this model. In murine platelets where thrombin induced PIP_2_ hydrolysis appears to be dependent on PIP5K1β but not PIP5Kγ [49]; since both these enzymes are expressed in platelets this implies the existence of two pools of PIP_2_ in these cells of which the PIP5K1β dependent pool is available for thrombin dependent PIP_2_ turnover. This finding together with our study in *Drosophila* photoreceptors implies that the plasma membrane in general may contain a specific pool of PIP_2_ dedicated for the use of receptor dependent PLC signalling and synthesized by a specific PIPK. It is possible that given the high rates of PLC activated PIP_2_ turnover at the plasma membrane (such as the microvillar membrane in photoreceptors) eukaryotic cells have evolved a mechanism to generate distinct PIP_2_ pool for this purpose so that other PIP_2_ dependent functions at the plasma membrane remain unaffected by ongoing receptor activated PIP_2_ hydrolysis. It is likely that dPIP5K and mammalian PIP5K1β represent PIP5K enzymatic activities required to support such a pool of PIP_2_ at the plasma membrane. It is presently unclear what properties might make dPIP5K more suitable for generating PIP_2_ in the context of receptor triggered PLC activity. One possibility is that the kinetic properties of the enzyme encoded by *dPIP5K* is distinct from that encoded by *sktl* allowing it to function in the context of high rates of PIP_2_ turnover. Alternatively (or additionally) within the nanoscale organization of the microvillar plasma membrane, it is possible that dPIP5K is segregated such that PIP_2_ generated by this enzyme is available within molecular distances of the phototransduction machinery. Interestingly, *Drosophila* photoreceptors contain within their microvillar membrane a macromolecular signalling complex organized by the PDZ domain protein INAD. It is presently not known if dPIP5K is part of a similar complex but the existence of such mechanisms has been previously shown for mammalian PIP5K1γ in the context of focal adhesion function [50,51]. Interestingly, it has been reported that the INAD protein complex that includes PLCβ is recruited to detergent resistant membranes during light stimulation [52] which themselves have been previously implicated in the formation of PIP_2_ microdomains and receptor activated PIP_2_ turnover [53][54]. It is possible that the two PIPKs, SKTL and dPIP5K show differential localization to such domains thus generating and segregating such pools of PIP_2_ and further studies in this direction are likely to provide insight into this issue. Nevertheless our study provided evidence for the concept of distinct PIPK enzymes as the basis for functionally distinct pools of PIP_2_ at the plasma membrane. Further analysis in this system is likely to reveal the molecular basis for the organization of PIP_2_ pools at cellular membranes.

## Materials and Methods

### Fly culture and genetics

Flies (*Drosophila melanogaster*) were reared on medium containing corn flour, sugar, yeast powder, and agar along with antibacterial and antifungal agents. Flies were maintained at 25°C and 50% relative humidity. There was no internal illumination within the incubator and the flies were subjected to light pulses of short duration only when the incubator door was opened. When required, flies were grown in an incubator with timed illumination from a white light source (Intensity mentioned in the figure legends of each experiment).

The wild-type strain was Red Oregon-R. The following fly alleles and insertions were obtained for the experiments described here: *so^D^, norpA^p24^* (Bloomington Stock Center), *sktl* ^Δ20^ (Hugo Bellen), *rdgB^9^* (R. C. Hardie, Cambridge University), *dPIP5K* overexpression line- GS200386 (DGRC-Kyoto).

### Generation of a dPIP5K antibody

In order to generate an antibody to dPIP5K, the antigenic fragment (an ∼ 250 amino acid unique sequence at the C-terminus of dPIP5K) was expressed as a recombinant protein and purified by affinity chromatography. Polyclonal antibodies were generated in rats using standard immunization protocols.

### Generation of *dPIP5K* knockout by homologous recombination

A knockout of dPIP5K was generated using ‘ends-out’ homologous recombination [20]. A 5.4 kb sequence of *dPIP5K* genomic sequence was used to generate the donor construct. It consisted of two pieces of genomic *dPIP5K* cloned as insert 1 (3.17 kb) and insert 2 (2.3 kb) separated by a marker gene *white* (Pw^+^) which was flanked by stop codons. These targeting sequences were cloned into the vector pW25 [55]. Transgenic flies carrying this construct were generated and used to perform homologous recombination as previously described [20]. Potential recombinants, which were mapped onto chromosome II, were subjected to molecular analyses using a PCR-based method. Finally, eight individual mutant alleles of *dPIP5K* (termed as PC4, PC5, PC8, PC18, PC30, PC33, PC60, and PC62) were confirmed and one of these *dPIP5K^18^* was characterized in detail and used in all experiments described in this study. Since homozygous mutants in *dPIP5K* are semi-lethal during pupal development we recombined *dPIP5K^18^* onto a chromosome with an FRT site at 42B. This allele was used to generate mosaic animals in which only adult retinae were homozygous mutant [21,22].

### *dPIP5K* genomic rescue fly

A BAC clone encompassing *dPIP5K*,CH321-03B05 (in *attB-Pacman-Cm^R^* vector) was obtained from p[acman] resource [56]. This BAC clone was 57.178 Kb long and included the *dPIP5K* gene with extended 5’ and 3’ regions having the promoter and most of the regulatory regions of the gene. The presence of *dPIP5K* in the clone was verified by PCR using specific primers. This clone was microinjected into embryos and inserted via ΦC31 integration into the VK22attP docking site in the fly genome to generate the wild-type *dPIP5K* genomic transgene {Bac[*dPIP5K*]}. Classical genetic crosses were used to move Bac[*dPIP5K*] into the *dPIP5K^18^* mutant background. Protein expression from Bac[*dPIP5K*] was verified using Western blotting using a dPIP5K specific antibody.

### Western immunoblotting

Heads from 1 day old flies were homogenized in 2× SDS-PAGE sample buffer followed by boiling at 95°C for 5 min. Samples were separated using SDS-PAGE and electro blotted onto nitrocellulose membrane (Hybond-C Extra; GE Healthcare) using semidry transfer assembly (Bio-Rad). Following blocking with 5% Blotto (Santa Cruz Biotechnology, CA), blots were incubated overnight at 4°C in appropriate dilutions of primary antibodies [anti-α-tubulin (1:5000 dilution; E7 DSHB), anti-Gαq (1:1000 dilution), anti-TRP (1:5000 dilution), anti-Rh1 (1:200,4C5 DSHB), anti-INAD (1:2000) and anti-NORPA (1:1000)]. Protein immunoreacted with the primary antibody was visualized after incubation in 1:10,000 dilution of appropriate secondary antibody coupled to horseradish peroxidase (Jackson Immuno Research Laboratories) for 2 h at room temperature. Blots were developed with ECL (GE Healthcare) and imaged using a LAS 4000 instrument (GE Healthcare).

### Optical neutralization and scoring retinal degeneration

Flies were immobilized on ice, decapitated using a blade and fixed on a glass slide using a drop of colorless nail varnish. It was imaged using 40× oil objective of Olympus BX43 microscope. Quantitation of degeneration was done as previously described [57]

### Isolation of pure retinal tissue

Pure preparations of retinal tissue were collected using previously described methods [58]. Briefly, 0- to 12-hr-old flies were snap-frozen in liquid nitrogen and dehydrated in acetone at −20°C for 48 hr. The acetone was then drained off and the retinae dried at room temperature. They were cleanly separated from the head at the level of the basement membrane using a scalpel blade.

### RNA extraction and QPCR

RNA was extracted from *Drosophila* head using TRIzol reagent (Invitrogen). Purified RNA was treated with amplification grade DNase I (Invitrogen). cDNA conversion was done using SuperScript II RNase H–Reverse Transcriptase (Invitrogen) and random hexamers (Applied Biosystems). Quantitative PCR (QPCR) was performed with the Applied Biosystem 7500 Fast Real Time PCR instrument. Primers were designed at the exon-exon junction following the parameters recommended for QPCR. Transcript levels of the ribosomal protein 49 (RP49) were used for normalization across samples. Three separate samples were collected from each genotype, and duplicate measures of each sample were conducted to ensure the consistency of the data. The primers used for QPCR were as follows:

RP49 fwd: CGGATCGATATGCTAAGCTGT;RP49 rev: GCGCTTGTTCGATCCGTA;
*dPIP4K* fwd: CATCCGTACGTTGTGGAGAG; *dPIP4K* rev: AGATCCACATCGTTGCTCAG;
*sktl* fwd: CTCATGTCCATGTGTGCGTC; *sktl* rev: TTAATGGTGCTCATCAGTG;
*dPIP5K* fwd: AGCAGAGAAAACCGCTTAGG; *dPIP5K* rev: GGCGATTCACTGACTTATTCC

### Subcellular fractionation

Fractionation was performed as described in [52]; in short frozen fly heads were homogenized in lysis buffer (20 mM Hepes, 30 mM NaCl, 5 mM EDTA) with protease inhibitors (Roche) at 4°C. Homogenate was centrifuged at 600×g for 3 min to remove chitonous material. The supernatant was spun at 55,000 rpm (ca. 100K g) for 30 minutes at 4°C (Beckman Ultracentriguge, Optima LE-80K ultracentrifuge, using a SW 50.1 rotor) to separate membrane fraction from cytosol. Equal volume of each fraction were subjected to SDS PAGE and analyzed by Western immunoblotting.

### Immunohistochemistry

For immunofluorescence, retinae from flies (0–12 hour post eclosion) were dissected under low red light in phosphate buffered saline (PBS) and then fixed in 4% paraformaldehyde in PBS with 1mg/ml saponin in fixing solution for 30 min at room temperature. Fixed eyes were washed 3 times in PBST (PBS with 0.3% TritonX-100) for 10 minutes. The tissues were then incubated in blocking solution [5% fetal bovine serum (FBS) in PBST] for 2 hours at room temperature, after which the tissues were incubated with primary antibodies diluted in blocking solution [anti-p-Moesin-1:200[29]], anti-Rh1–1:50 (4C5, Developmental Studies Hybridoma Bank), anti-TRP-1:250,anti-dPIP5K-1:100, anti-GFP-1: 200 (Abcam)] overnight at 4°C. Appropriate secondary antibodies conjugated with a fluorophore were used at 1:300 dilutions [Alexa Fluor 633/568 IgG, (Molecular Probes)] and incubated for 2 hours at room temperature. Wherever required, during the incubation with secondary antibody, Alexafluor 568–phalloidin (Invitrogen) was also added to the tissues to stain the F-actin. After three washes in PBST, the tissues were washed in PBS for 10 min, mounted in mounting medium (70% glycerol in PBS). The whole-mounted preparations were viewed under Olympus FV1000 laser scanning confocal microscope.

### Electroretinogram recordings

Flies were anesthetized and immobilized at the end of a disposable pipette tip using a drop of low melt wax. Recordings were done using glass microelectrodes filled with 0.8% w/v NaCl solution. Voltage changes were recorded between the surface of the eye and an electrode placed on the thorax. Following fixing and positioning, flies were dark adapted for 6 min. ERG was recorded with 2 second flashes of green light stimulus. Stimulating light was delivered from a LED light source to within 5 mm of the fly’s eye through a fiber optic guide. Calibrated neutral density filters were used to vary the intensity of the light source. Voltage changes were amplified using a DAM50 amplifier (WPI) and recorded using pCLAMP 10.2. Analysis of traces was performed using Clampfit (Axon Laboratories).

### PIP_2_ dynamics

To monitor PIP_2_ dynamics in live flies, transgenic flies expressing PH-PLCδ::GFP (PIP_2_ biosensor) were anesthetized and immobilized at the end of a pipette tip using a drop of low melt wax and fixed by clay on the stage of an Olympus IX71 microscope. The fluorescent deep pseudopupil (dpp, a virtual image that sums rhabdomere fluorescence from ∼20–40 adjacent ommatidia) was focused and imaged using a 10× objective. Time-lapse images were taken by exciting GFP using a 90ms flash of blue light and collecting emitted fluorescence. The program used for this purpose was created in Micromanager. Following preparation, flies were dark adapted for seven minutes after which the eye was stimulated with a 90 ms flash of blue light. The blue light used to excite GFP was also the stimulus to rapidly convert the majority of rhodopsin (R) to metarhodopsin (M) thus activating the phototransduction cascade and triggering depletion of rhabdomeric PIP_2_. Between the blue light stimulations, photoreceptors were exposed to long wavelength (red) light that reconverts M to R. The resurgence in dpp fluorescence with time indicates translocation of the probe from cytoplasm to rhabdomere membrane upon PIP_2_ re-synthesis. The dpp intensity was measured using ImageJ from NIH (Bethesda, MD, USA). Cross sectional areas of rhabdomeres of R1-R6 photoreceptors were measured and the mean intensity values per unit area were calculated.

### Electron microscopy

Samples for TEM were prepared as mentioned in Ref.^44^. Briefly eyes were bisected in ice-cold fixative (2.5% glutaraldehyde in 0.1 M PIPES buffer [pH 7.4]). After 10hrs of fixation at 4°C, eyes were washed with 0.1M PIPES, post-fixed in 1% OsO_4_ (30min), and stained en bloc in 2% uranyl acetate (1 hr). Eyes were dehydrated in ethanol series and embedded in epoxy. Ultrathin sections (50 nm) were cut and viewed on a Tecnai G2 Spirit Bio-TWIN electron microscope.

## Supporting Information

S1 Fig Analysis of *sktl* in *Drosophila* photoreceptors. (A) Representative light responses from *sktl^Δ20^*/+ and control flies of matched eye color. (B) ERG responses from control (Rh1-Gal4/+) and flies expressing *sktl*-kinase dead (K/D) using Rh1-Gal4. (C) Confocal image showing localization of SKTL::RFP expressed using Rh1-Gal4 in adult *Drosophila* photoreceptors. Retinae were labeled with an antibody to Rh1. Co-localization of both proteins to the microvilli is shown. (D) Quantification of PIP_2_ dynamics in flies overexpressing *sktl* compared to controls. X-axis represents time in minutes and Y-axis represents the level of fluorescence represented as a % of the value in the initial image. Error bars represents mean +/− S.D from five flies.
(TIF)
