# Supplementary figures and images for: A dPIP5K Dependent Pool of Phosphatidylinositol 4,5 Bisphosphate (PIP2) Is Required for G-Protein Coupled Signal Transduction in Drosophila Photoreceptors
Source: PLoS Genet. 2015 Jan 29;11(1):e1004948. doi: 10.1371/journal.pgen.1004948 (PMC4310717; doi:10.1371/journal.pgen.1004948)

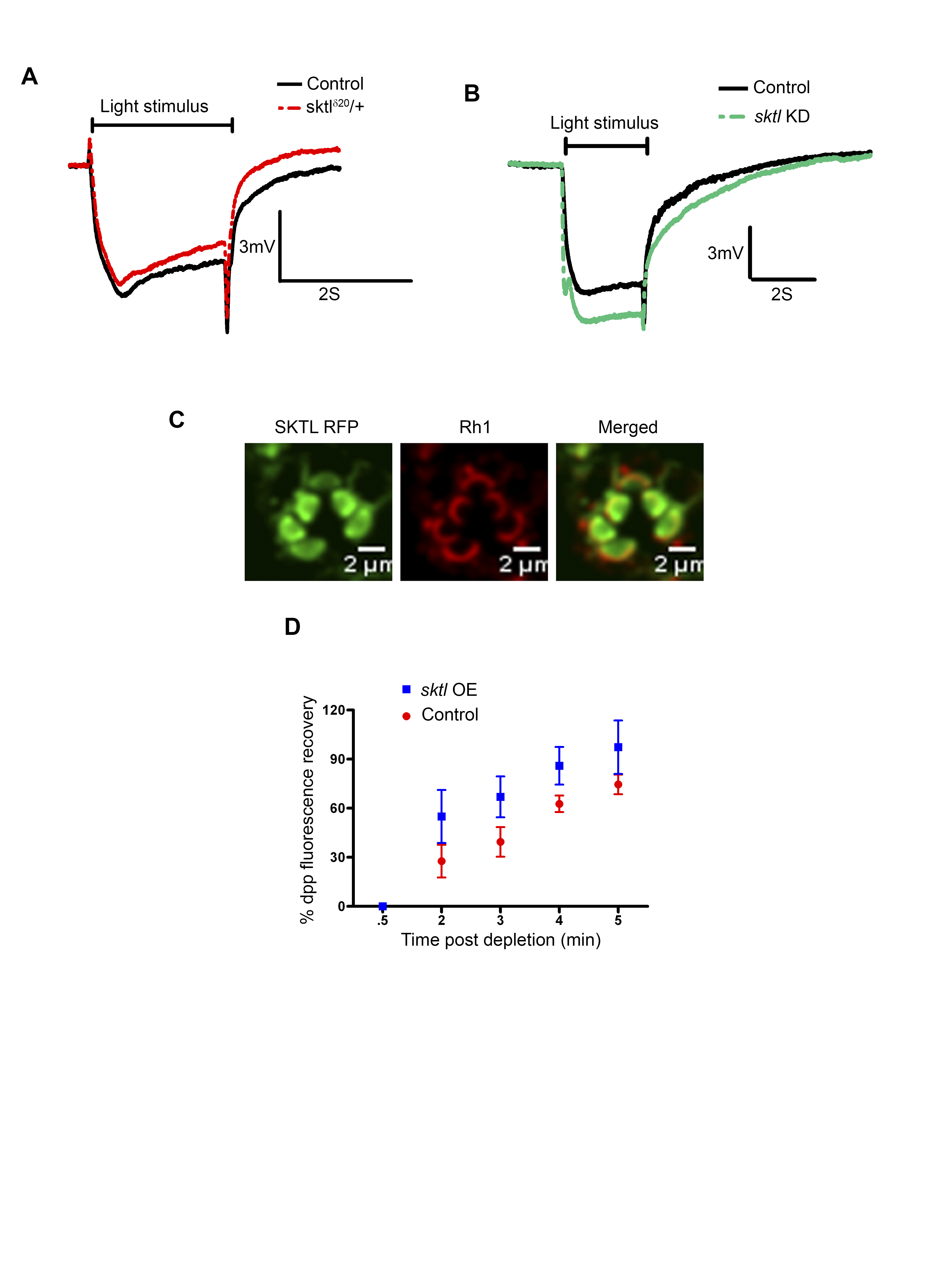

Supplement: S1 Fig — (A) Representative light responses from sktlΔ20/+ and control flies of matched eye color. (B) ERG responses from control (Rh1-Gal4/+) and flies expressing sktl-kinase dead (K/D) using Rh1-Gal4. (C) Confocal image showing localization of SKTL::RFP expressed using Rh1-Gal4 in adult Drosophila photoreceptors. Retinae were labeled with an antibody to Rh1. Co-localization of both proteins to the microvilli is shown. (D) Quantification of PIP2 dynamics in flies overexpressing sktl compared to controls. X-axis represents time in minutes and Y-axis represents the level of fluorescence represented as a % of the value in the initial image. Error bars represents mean +/− S.D from five flies. (TIF) [file pgen.1004948.s001.tif]
